# Spinal Atomic Lambda-Calculus

**DOI:** 10.1007/978-3-030-45231-5_30

**Published:** 2020-04-17

**Authors:** David Sherratt, Willem Heijltjes, Tom Gundersen, Michel Parigot

**Affiliations:** 8 ENS/CNRS, Université Paris-Saclay, Cachan, France; 9 University of Duisburg-Essen, Duisburg, Germany; 10 Friedrich-Schiller-Universität Jena, Jena, Germany; 11 University of Bath, Bath, UK; 12 Red Hat, Inc., Oslo, Norway; 13 Institut de Recherche en Informatique Fondamentale, CNRS, Université de Paris, Paris, France

**Keywords:** Lambda-Calculus, Full laziness, Deep inference, Curry–Howard

## Abstract

We present the spinal atomic $\lambda$-calculus, a typed $\lambda$-calculus with explicit sharing and atomic duplication that achieves spinal full laziness: duplicating only the direct paths between a binder and bound variables is enough for beta reduction to proceed. We show this calculus is the result of a Curry–Howard style interpretation of a deep-inference proof system, and prove that it has natural properties with respect to the $\lambda$-calculus: confluence and preservation of strong normalisation.

## 1 Introduction

In the λ-calculus, a main source of efficiency is *sharing*: multiple use of a single subterm, commonly expressed through graph reduction [27] or explicit substitution [1]. This work, and the *atomic λ-calculus* [16] on which it builds, is an investigation into sharing as it occurs naturally in intuitionistic *deep-inference* proof theory [26]. The atomic λ-calculus arose as a Curry–Howard interpretation of a deep-inference proof system, in particular of the *distribution* rule given below left, a variant of the characteristic *medial* rule [10, 26]. In the term calculus, the corresponding *distributor* enables duplication to proceed *atomically*, on individual constructors, in the style of sharing graphs [21]. As a consequence, the natural reduction strategy in the atomic λ-calculus is *fully lazy* [27, 4]: it duplicates only the minimal part of a term, the *skeleton*, that can be obtained by lifting out subterms as explicit substitutions. (While duplication is atomic *locally*, a duplicated abstraction does not form a redex until also its bound variables have been duplicated; hence duplication becomes fully lazy *globally*.)

This work was supported by EPSRC Project EP/R029121/1 *Typed Lambda-Calculi with Sharing and Unsharing* and ANR project 15-CE25-0014 *The Fine Structure of Formal Proof Systems and their Computational Interpretations (FISP)*

Distribution: $\dfrac{A \to (B \wedge C)}{(A \to B) \wedge (A \to C)}\, d$ Switch: $\dfrac{(A \to B) \wedge C}{A \to (B \wedge C)}\, s$

We investigate the computational interpretation of another characteristic deep-inference proof rule: the *switch* rule above right [26].[5] Our result is the *spinal atomic λ-calculus*, a λ-calculus with a refined form of full laziness, *spine duplication*. In the terminology of [4], this strategy duplicates only the *spine* of an abstraction: the paths to its bound variables in the syntax tree of the term.[6]

We illustrate these notions in Figure 1, for the example $\lambda x.\lambda y.((\lambda z.z)y)x$. The *scope* of the abstraction $\lambda x$ is the entire subterm, $\lambda y.((\lambda z.z)y)x$ (which may or may not be taken to include $\lambda x$ itself). Note that with explicit substitution, the scope may grow or shrink by lifting explicit substitutions in or out. The *skeleton* is the term $\lambda x.\lambda y.(wy)x$ where the subterm $\lambda z.z$ is lifted out as an (explicit) substitution $[\lambda z.z/w]$. The *spine* of a term, indicated in the second image, cannot naturally be expressed with explicit substitution, though one can get an impression with *capturing* substitutions: it would be $\lambda x.\lambda y.wx$, with the subterm $(\lambda z.z)y$ extracted by a capturing substitution $[(\lambda z.z)y/w]$. Observe that the skeleton can be described as the *iterated spine*: it is the smallest subgraph of the syntax tree closed under taking the spine of each abstraction, i.e. that contains the spine of every abstraction it contains.

These notions give rise to four natural duplication regimes. For a shared abstraction to become available as the function in a $\beta$-redex: *laziness* duplicates its *scope* [22]; *Full laziness* duplicates its *skeleton* [27]; *Spinal full laziness* duplicates its *spine* [8]; *optimal reduction* duplicates only the abstraction $\lambda x$ and its bound variables $x$ [21, 3].[7]

While each of these duplication strategies has been expressed in graphs and labelled calculi, the atomic λ-calculus is the first term calculus with Curry–Howard corresponding proof system to naturally describe full laziness. Likewise, the spinal atomic λ-calculus presented here is the first term calculus with Curry–Howard corresponding proof system to naturally describe spinal full laziness.

*Switch and Spine.* One way to describe the skeleton or the spine of an abstraction within a λ-term is through explicit end-of-scope markers, as explored by Berkling and Fehr [7], and more recently by Hendriks and Van Oostrom [18]. We use their *adbmal* (ʎ) to illustrate the idea: the constructor $ʎx.N$ indicates that the subterm $N$ does not contain occurrences of $x$ (or that any that do occur are

[5] The switch rule is an intuitionistic variant of *weak* or *linear distributivity* [12] for multiplicative linear logic.

[6] There is a clash of (existing) terminology: the *spine of an abstraction*, as we use here, is a different notion from the *spine of a λ-term*, which is the path from the root to the leftmost variable, as used e.g. in head reduction and abstract machines.

[7] Interestingly, Balabonski [5] shows that for *weak* reduction (where one does not reduce under an abstraction) full laziness and spinal full laziness are both optimal (in the number of beta-steps required to reach a normal form).

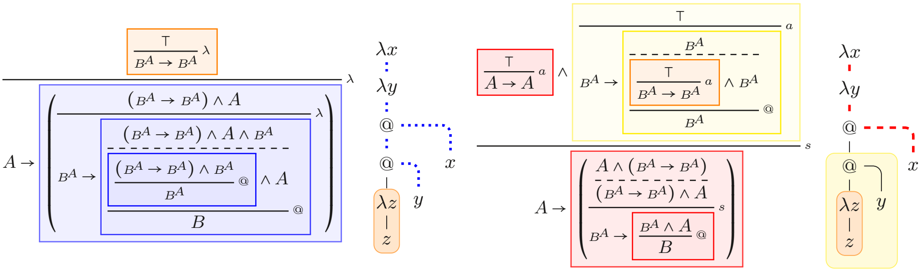

Fig. 1: Balanced and unbalanced typing derivations for $\lambda x.\lambda y.((\lambda z.z)y)x$, with corresponding graphical representations of the term. The variable $x$ has type $A$ and $y, z$ type $A \to B$, shortened to $B^A$. The left derivation isolates the skeleton of $\lambda x$, and the right derivation its spine, both by the subderivations in braces.

not available to a binder $\lambda x$ outside $ʎx.N$). The scope of an abstraction thus becomes explicitly indicated in the term. This opens up a distinction between *balanced* and *unbalanced* scopes: whether scopes must be properly nested, or not; for example, in $\lambda x.\lambda y.N$, a subterm $ʎy.ʎx.M$ is balanced, but $ʎx.ʎy.M$ is not. With balanced scope, one can indicate the skeleton of an abstraction; with unbalanced scope (which Hendriks and Van Oostrom dismiss) one can indicate the spine. We do so for our example term $\lambda x.\lambda y.((\lambda z.z)y)x$ below.

| | |
|---|---|
| Balanced scope/skeleton: | $\lambda x.\lambda y.(ʎy.(ʎx.\lambda z.z)y)(ʎy.x)$ |
| Unbalanced scope/spine: | $\lambda x.\lambda y.(ʎx.(ʎy.\lambda z.z)y)(ʎy.x)$ |

A closely related approach is *director strings*, introduced by Kennaway and Sleep [19] for combinator reduction and generalized to any reduction strategy by Fernández, Mackie, and Sinot in [13]. The idea is to use nameless abstractions identified by their nesting (as with De Bruijn indices), and make the paths to bound variables explicit by annotating each constructor with a string of *directors*, that outline the paths. The primary aim of these approaches is to eliminate $\alpha$-conversion and to streamline substitution. Consequently, while they can *identify* the spine, they do not readily isolate it for duplication.

The present work starts from our observation that the *switch* rule of open deduction functions as a proof-theoretic end-of-scope construction (see [25] for details). However, it does so in a *structural* way: it forces a deconstruction of a proof into readily duplicable parts, which together may form the spine of an abstraction. The derivations in Figure 1 demonstrate this, as we will now explain—see the next section for how they are formally constructed.

The abstraction $\lambda x$ corresponds in the proof system to the implication $A\to$, explicitly scoping over its right-hand side. On the left, with the *abstraction* rule ($\lambda$), scopes must be balanced, and the proof system may identify the *skeleton*; here, that of $\lambda x$ as the largest blue box. Decomposing the abstraction ($\lambda$) into *axiom* ($a$) and *switch* ($s$), on the right the proof system may express unbalanced scope. It does so by separating the scope of an abstraction into multiple parts; here, that of $\lambda x$ is captured as the two top-level red boxes. Each box is ready to be duplicated; in this way, one may duplicate the spine of an abstraction only.

These two derivations correspond to terms in our calculus. The subterms not part of the skeleton (i.e. $\lambda z.z$) remain shared and we are able to duplicate the skeleton alone. This is also possible in [16]. In our calculus we are also able to duplicate just the spine by using a *distributor*. We require this construct as otherwise we break the binding of the $y$-abstraction. The distributor manages and maintains these bindings. The $y$-abstraction in the spine ($y\langle a\rangle$) is a *phantom-abstraction*, because it is not real and we cannot perform $\beta$-reduction on it. However, it may become real during reduction. It can be seen as a placeholder for the abstraction. The variables in the *cover* ($a$) represent subterms that both remain shared and are found in the distributor.

$$\text{Skeleton:} \quad \underline{\lambda x.\lambda y.(a\,y)\,x}\,[a \leftarrow \lambda z.z]$$

$$\text{Spine:} \quad \underline{\lambda x.y\langle a\rangle.(a)\,x}\,[y\langle a\rangle \,|\, \lambda y.\,[a \leftarrow (\lambda z.z)y]]$$

Our investigation is then focused on the interaction of switch and distribution (later observed in the rewrite rule $l_5$). The use of the distribution rule allows us to perform duplication atomically, and thus provides a natural strategy for spinal full laziness. In Figure 1 on the right, this means duplicating the two top-level red boxes can be done independently from duplicating the yellow box.

## 2 Typing a $\lambda$-calculus in open deduction

We work in *open deduction* [15], a formalism of deep-inference proof theory, using the following proof system for (conjunction–implication) intuitionistic logic. A *derivation* from a *premise* formula $X$ to a *conclusion* formula $Z$ is constructed inductively as in Figure 2a, with from left to right: a propositional atom $a$, where $X = Z = a$; *horizontal composition* with a connective $\rightarrow$, where $X = Y \rightarrow X_2$ and $Z = Y \rightarrow Z_2$; *horizontal composition* with a connective $\wedge$, where $X = X_1 \wedge X_2$ and $Z = Z_1 \wedge Z_2$; and *rule composition*, where $r$ is an inference rule (Figure 2b) from $Y_1$ to $Y_2$. The boxes serve as parentheses (since derivations extend in two dimensions) and may be omitted. Derivations are considered up to associativity of rule composition. One may consider formulas as derivations that omit rule composition. We work modulo associativity, symmetry, and unitality of conjunction, justifying the $n$-ary contraction, and may omit $\top$ from the axiom rule. A 0-ary contraction, with conclusion $\top$, is a *weakening*. Figure 2b: the abstraction rule ($\lambda$) is derived from axiom and switch. *Vertical composition* of a derivation from $X$ to $Y$ and one from $Y$ to $Z$, depicted by a dashed line, is a defined operation, given in Figure 2c, where $\ast \in \{\wedge, \rightarrow\}$.

### 2.1 The Sharing Calculus

Our starting point is the *sharing calculus* ($\Lambda^S$), a calculus with an explicit sharing construct, similar to explicit substitution.

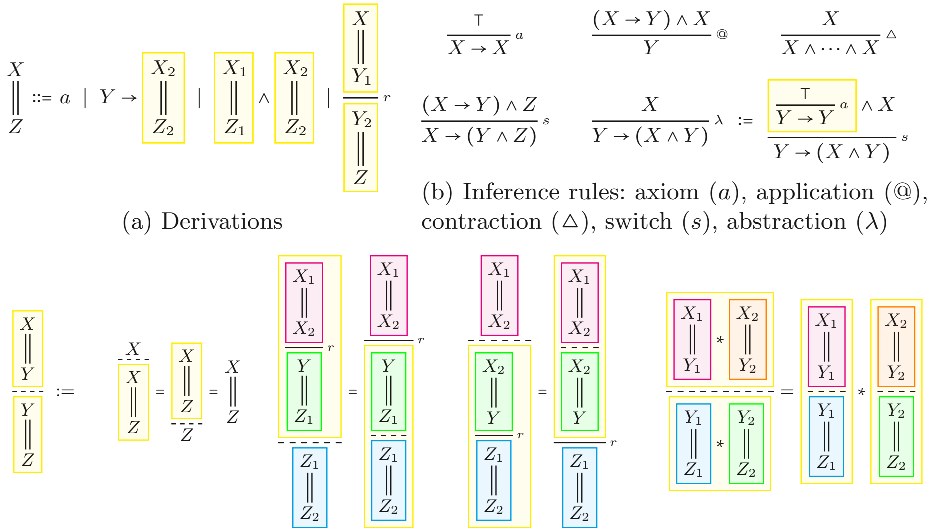

(a) Derivations

(b) Inference rules: axiom ($a$), application (@), contraction ($\triangle$), switch ($s$), abstraction ($\lambda$)

(c) Vertical composition

Fig. 2: Intuitionistic proof system in open deduction

**Definition 1.** *The **pre-terms** $r, s, t, u$ and **sharings** $[\Gamma]$ of the $\Lambda^S$ are defined by:*

$$s, t ::= x \mid \lambda x.t \mid s\,t \mid t[\Gamma] \qquad [\Gamma] ::= [x_1, \dots, x_n \leftarrow s]$$

*with from left to right: a **variable**; an **abstraction**, where $x$ occurs free in $t$ and becomes bound; an **application**, where $s$ and $t$ use distinct variable names; and a **closure**; in $t[\vec{x} \leftarrow s]$ the variables in the vector $\vec{x} = x_1, \dots, x_n$ all occur in $t$ and become bound, and $s$ and $t$ use distinct variable names. **Terms** are pre-terms modulo **permutation** equivalence ($\sim$):*

$$t[\vec{x} \leftarrow s][\vec{y} \leftarrow r] \sim t[\vec{y} \leftarrow r][\vec{x} \leftarrow s] \qquad (\{\vec{y}\} \cap (s)_{fv} = \{\})$$

*A term is in **sharing normal form** if all sharings occur as $[\vec{x} \leftarrow x]$ either at the top level or directly under a binding abstraction, as $\lambda x.t[\vec{x} \leftarrow x]$.*

Note that variables are *linear*: variables occur at most once, and bound variables must occur. A vector $\vec{x}$ has length $|\vec{x}|$ and consist of the variables $x_1, \dots, x_{|\vec{x}|}$. An **environment** is a sequence of sharings $\overline{[\Gamma]} = [\Gamma_1] \dots [\Gamma_n]$. Substitution is written $\{t/x\}$, and $\{t_1/x_1\} \dots \{t_n/x_n\}$ may be abbreviated to $\{t_i/x_i\}_{i \in [n]}$.

**Definition 2.** *The **interpretation** $[\![ - ]\!] : \Lambda \to \Lambda^S$ is defined below.*

$$[\![ x ]\!] = x \quad [\![ \lambda x.t ]\!] = \lambda x.[\![ t ]\!] \quad [\![ s\,t ]\!] = [\![ s ]\!]\,[\![ t ]\!] \quad [\![ t[\vec{x} \leftarrow s] ]\!] = [\![ t ]\!]\{[\![ s ]\!]/x_i\}_{i \in [n]}$$

The **translation** $(\!| N |\!)$ of a $\lambda$-term $N$ is the unique sharing-normal term $t$ such that $N = [\![ t ]\!]$. A term $t$ will be typed by a derivation with restricted types,

Basic Types: $A, B, C := a \mid A \to B$ Context Types: $\Gamma, \Delta, \Omega := A \mid \top \mid \Gamma \wedge \Delta$

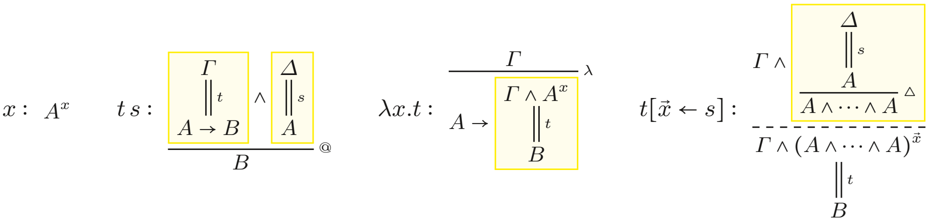

Fig. 3: Typing system for $\Lambda^S$

as shown below, where the *context type* $\Gamma = A_1 \wedge \cdots \wedge A_n$ will have an $A_i$ for each free variable $x_i$ of $t$. We connect free variables to their premises by writing $A^x$ and $\Gamma^{\vec{x}}$. The $\Lambda^S$ is then typed as in Figure 3.

## 3 The Spinal Atomic λ-Calculus

We now formally introduce the syntax of the spinal atomic λ-calculus ($\Lambda_a^S$), by extending the definition of the sharing calculus in Definition 1 with a *distributor* construct that allows for atomic duplication of terms.

**Definition 3 (Pre-Terms).** *The* ***pre-terms*** $r, s, t$*,* ***closures*** $[\Gamma]$*, and* ***environments*** $\overline{[\Gamma]}$ *of the* $\Lambda_a^S$ *are defined by:*

$$t \ ::= \ x \ \mid \ s\,t \ \mid \ x\langle\,\vec{y}\,\rangle.t \ \mid \ t[\Gamma] \qquad \overline{[\Gamma]} \ ::= \ [\Gamma] \ \mid \ \overline{[\Gamma]}[\Gamma]$$
$$[\Gamma] \ ::= \ [\vec{x} \leftarrow t] \ \mid \ [\vec{x}\,|\,y\langle\,\vec{z}\,\rangle\,\overline{[\Gamma]}]$$

Our generalized abstraction $x\langle\,\vec{y}\,\rangle.t$ is a **phantom-abstraction**, where $x$ a **phantom-variable** and the **cover** $\vec{y}$ will be a subset of the free variables of $t$. It can be thought of as a "delayed" abstraction: $x$ is a binder, but possibly not in $t$ itself, and instead in the terms substituted for the variables $\vec{y}$; in other words, $x$ is a *capturing* binder for substitution into $\vec{y}$. We define standard λ-abstraction as the special case $\lambda x.t \equiv x\langle\,x\,\rangle.t$, and generally, when we refer to $x\langle\,\vec{y}\,\rangle$ as a phantom-abstraction (rather than an abstraction) we assume $\vec{y} \neq x$. The **distributor** $u[\vec{x}\,|\,y\langle\,\vec{z}\,\rangle\,\overline{[\Gamma]}]$ binds the phantom-variables $\vec{x}$ in $u$, while its environment $\overline{[\Gamma]}$ will bind the variables in their covers; intuitively, it represents a set of explicit substitutions in which the variables $\vec{x}$ are expected to be captured.

The distributor is introduced when we wish to duplicate an abstraction, as depicted in Figure 4a. The sharing node (∘) duplicates the abstraction node, creating a distributor (depiced as the sharing and unsharing node (•), together with the bindings of the phantom-variables (depicted with a dashed line). The variables captured by the environment are the variables connected to sharing nodes linked with a dotted line. Notice one sharing node can be linked with multiple unsharing nodes, and vice versa. Duplication of applications also duplicates

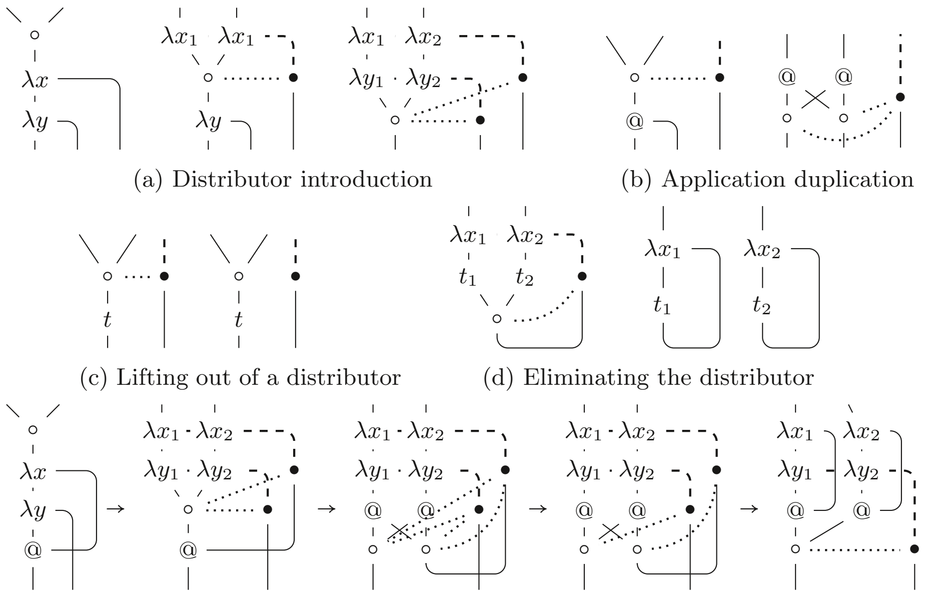

(a) Distributor introduction (b) Application duplication
(c) Lifting out of a distributor (d) Eliminating the distributor
(e) Duplicating the spine

Fig. 4: Graphical illustration of the distributor the dotted line (Figure 4b), but these can be removed later if the term does not contain the variable bound to the unsharing (Figure 4c). These subterms are those which are not part of the spine. Eventually, we will reach a state where the only sharing node connected to the unsharing node is the one that shared the variable bound to the unsharing, allowing us to eliminate the distributor (Figure 4d). The purpose of the dotted line is similar to the brackets of optimal reduction graphs [21, 24], to supervise which sharing and unsharing match.

Terms are then pre-terms with sensible and correct bindings. To define terms, we first define *free* and *bound* variables and phantom variables; variables are bound by abstractions (not phantoms) and by sharings, while phantom-variables are bound by distributors.

**Definition 4 (Free and Bound Variables).** *The **free variables** $(-)_{fv}$ and **bound variables** $(-)_{bv}$ of a pre-term $t$ are defined as follows*

$$
\begin{aligned}
(x)_{fv} &= \{x\} & (x)_{bv} &= \{\} \\
(s\,t)_{fv} &= (s)_{fv} \cup (t)_{fv} & (s\,t)_{bv} &= (s)_{bv} \cup (t)_{bv} \\
(x\langle\, x\,\rangle.t)_{fv} &= (t)_{fv} - \{x\} & (x\langle\, x\,\rangle.t)_{bv} &= (t)_{bv} \cup \{x\} \\
(x\langle\, \vec{y}\,\rangle.t)_{fv} &= (t)_{fv} & (x\langle\, \vec{y}\,\rangle.t)_{bv} &= (t)_{bv} \\
(u[\vec{x} \leftarrow t])_{fv} &= (u)_{fv} \cup (t)_{fv} - \{\vec{x}\} & (u[\vec{x} \leftarrow t])_{bv} &= (u)_{bv} \cup (t)_{bv} \cup \{\vec{x}\} \\
(u[\vec{x}\,|\,y\langle\, y\,\rangle\,\overline{[\Gamma]}])_{fv} &= (u\overline{[\Gamma]})_{fv} - \{y\} & (u[\vec{x}\,|\,y\langle\, y\,\rangle\,\overline{[\Gamma]}])_{bv} &= (u\overline{[\Gamma]})_{bv} \cup \{y\}
\end{aligned}
$$

$$(u[\vec{x}\,|\,y\langle\,\vec{z}\,\rangle\,\overline{[\Gamma]}])_{fv} = (u\overline{[\Gamma]})_{fv} \cup \{y\} \qquad (u[\vec{x}\,|\,y\langle\,\vec{z}\,\rangle\,\overline{[\Gamma]}])_{bv} = (u\overline{[\Gamma]})_{bv}$$

**Definition 5 (Free and Bound Phantom-Variables).** *The* ***free phantom-variables*** $(-)_{fp}$ *and* ***bound phantom-variables*** $(-)_{bp}$ *of the pre-term $t$ are defined as follows*

$$\begin{aligned}
(x)_{fp} &= \{\} & (x)_{bp} &= \{\}\\
(s\,t)_{fp} &= (s)_{fp} \cup (t)_{fp} & (s\,t)_{bp} &= (s)_{bp} \cup (t)_{bp}\\
(x\langle\,x\,\rangle.t)_{fp} &= (t)_{fp} & &\\
(c\langle\,\vec{x}\,\rangle.t)_{fp} &= (t)_{fp} \cup \{c\} & (c\langle\,\vec{x}\,\rangle.t)_{bp} &= (t)_{bp}\\
(u[\vec{x} \leftarrow t])_{fp} &= (u)_{fp} \cup (t)_{fp} & (u[\vec{x} \leftarrow t])_{bp} &= (u)_{bp} \cup (t)_{bp}\\
(u[\vec{x}\,|\,c\langle\,c\,\rangle\,\overline{[\Gamma]}])_{fp} &= (u\overline{[\Gamma]})_{fp} - \{\vec{x}\} & &\\
(u[\vec{x}\,|\,c\langle\,\vec{y}\,\rangle\,\overline{[\Gamma]}])_{fp} &= (u\overline{[\Gamma]})_{fp} \cup \{c\} - \{\vec{x}\} & (u[\vec{x}\,|\,c\langle\,\vec{y}\,\rangle\,\overline{[\Gamma]}])_{bp} &= (u\overline{[\Gamma]})_{bp} \cup \{\vec{x}\}
\end{aligned}$$

The **free covers** $(u)_{fc}$ and **bound covers** $(u)_{bc}$ are the covers associated with the free phantom-variables $(u)_{fp}$ respectively the bound phantom-variables $(u)_{bp}$ of $u$; that is, if $x$ occurs as $x\langle\,\vec{a}\,\rangle$ in $u$ and $x \in (u)_{fp}$ then $\langle\vec{a}\rangle \in (u)_{fc}$. When bound, $x$ and the variables in $\vec{a}$ may be alpha-converted independently. When a distributor $u[\vec{x}\,|\,y\langle\,\vec{z}\,\rangle\,\overline{[\Gamma]}]$ binds the phantom-variables $\vec{x} = x_1, \ldots, x_n$ where each $x_i$ occurs as $x_i\langle\,\vec{a}_i\,\rangle$ in $u$, then for technical convenience we may make the covers explicit in the distributor itself, and write

$$u[x_1\langle\,\vec{a}_1\,\rangle \ldots x_n\langle\,\vec{a}_n\,\rangle\,|\,y\langle\,\vec{z}\,\rangle\,\overline{[\Gamma]}]\ .$$

The environment $\overline{[\Gamma]}$ is expected to bind *exactly* the variables in the covers $\langle\vec{a}_i\rangle$. We apply this and other restrictions to define the terms of the calculus.

**Definition 6.** ***Terms*** $t \in \Lambda_a^S$ *are pre-terms with the following constraints*

1. *Each variable may occur at most once.*
2. *In a phantom-abstraction* $x\langle\,\vec{y}\,\rangle.t$, $\{\vec{y}\} \subseteq (t)_{fv}$.
3. *In a sharing* $u[\vec{x} \leftarrow t]$, $\{\vec{x}\} \subseteq (u)_{fv}$.
4. *In a distributor* $u[x_1\langle\,\vec{a}_1\,\rangle \ldots x_n\langle\,\vec{a}_n\,\rangle\,|\,y\langle\,\vec{z}\,\rangle\,\overline{[\Gamma]}]$
   (a) $\{x_1, \ldots, x_n\} \subseteq (u)_{fp}$;
   (b) *the variables in* $\bigcup_{i \leq n}\{\vec{a}_i\}$ *are free in $u$ and bound by* $\overline{[\Gamma]}$.
   (c) *the variables in* $\{\vec{z}\}$ *occur freely in the environment* $\overline{[\Gamma]}$.

*Example 1.* Here we show some pre-terms that are not terms.

- $c\langle\,x\,\rangle.y$ (violates condition 2)
- $x\,y[x, z \leftarrow w]$ (violates condition 3)
- $e_2\langle\,w_2\,\rangle.w_2\,((e_1\langle\,w_1\,\rangle.w_1)\,z)[e_1\langle\,w_1\,\rangle, e_2\langle\,w_2\,\rangle\,|\,c\langle\,z\,\rangle\,[w_1, w_2 \leftarrow x\langle\,x\,\rangle.x\,y]]$ (violates condition $4a$)

We also work modulo permutation with respect to the variables in the cover of phantom-abstractions. Let $\vec{x}$ be a list of variables and let $\vec{x_P}$ be a permutation of that list, then the following terms are considered equal.

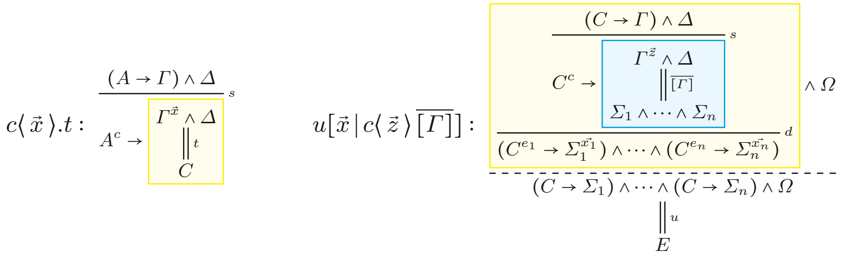

Fig. 5: Typing derivations for phantom-abstractions and distributors

$$u[\vec{x} \leftarrow t] \sim u[\vec{x_P} \leftarrow t] \qquad\qquad y\langle\, \vec{x}\,\rangle.t \sim y\langle\, \vec{x_P}\,\rangle.t$$

Terms are typed with the typing system for $\Lambda^S$ extended with the *distribution* inference rule. This rule is the result of computationally interpreting the medial rule as done in [16]. We obtain this variant of the medial rule due to the restriction for implications and to avoid introducing disjunction to the typing system. The terms of $\Lambda^S_a$ are then typed as in both Figure 3 and Figure 5. Note environments are typed by the derivations of all its closures composed horizontally with the conjunction connective. Also note that in the case for phantom-abstraction is similar for that of an abstraction, where we replace one occurrence of the simple type $A$ by the conjunction $\Gamma$.

### 3.1 Compilation and Readback.

We now define the translations between $\Lambda^S_a$ and the original $\lambda$-calculus. First we define the interpretation $\Lambda \to \Lambda^S_a$ (*compilation*). Intuitively, it replaces each abstraction $\lambda x.-$ with the term $x\langle\, x\,\rangle.-[x_1, \ldots, x_n \leftarrow x]$ where $x_1, \ldots, x_n$ replace the occurrences of $x$. Actual substitutions are denoted as $\{t/x\}$. Let $|\,M\,|_x$ denote the number of occurrences of $x$ in $M$, and if $|\,M\,|_x = n$ let $M\frac{n}{x}$ denote $M$ with the occurrences of $x$ replaced by fresh, distinct variables $x_1, \ldots, x_n$. First, the translation of a *closed* term $M$ is $(\!|\, M \,|\!)'$, defined below

**Definition 7 (Compilation).** *The interpretation of* $\lambda$ *terms,* $(\!|\, \Lambda \,|\!)' : \Lambda \to \Lambda^S_a$*, is defined as*

$$(\!|\, M\frac{n_1}{x_1} \ldots \frac{n_k}{x_k} \,|\!)'[x_1^1, \ldots, x_1^{n_1} \leftarrow x_1] \ldots [x_k^1, \ldots, x_k^{n_k} \leftarrow x_k]$$

*where* $x_1, \ldots, x_k$ *are the free variables of* $M$ *such that* $|\,M\,|_{x_i} = n_i > 1$ *and* $(\!|\, - \,|\!)'$ *is defined on terms as (where* $n \neq 1$ *in the abstraction case):*

$$(\!|\, x \,|\!)' = x \qquad (\!|\, M\,N \,|\!)' = (\!|\, M \,|\!)'\,(\!|\, N \,|\!)' \qquad (\!|\, \lambda x.M \,|\!)' = \begin{cases} x\langle\, x\,\rangle.(\!|\, M \,|\!)' & \text{if } |\,M\,|_x = 1 \\ x\langle\, x\,\rangle.(\!|\, M\frac{n}{x} \,|\!)'[x_1, \ldots, x_n \leftarrow x] & \text{if } |\,M\,|_x = n \end{cases}$$

The readback into the $\lambda$-calculus is slightly more complicated, specifically due to the bindings induced by the distributor. Interpreting a distributor construct as a $\lambda$-term requires (1) converting the phantom-abstractions it binds in

$u$ into abstractions (2) collapsing the environment (3) maintaining the bindings between the converted abstractions and the intended variables located in the environment.

**Definition 8.** *Given a total function $\sigma$ with domain $D$ and codomain $C$, we* ***overwrite*** *the function with case $x \mapsto v$ where $x \in D$ and $v \in C$ such that*

$$\sigma[x \mapsto v](z) \quad := \quad \textit{if } (x = z) \textit{ then } v \textit{ else } \sigma(z)$$

We use the map $\sigma$ as part of the translation, the intuition is that for all bound variables $x$ in the term we are translating, it should be that $\sigma(x) = x$. The purpose of the map $\gamma$ is to keep track of the binding of phantom-variables.

**Definition 9.** *The interpretation $[\![ - | - | - ]\!] : \Lambda_a^S \times (V \to \Lambda) \times (V \to V) \to \Lambda$ is defined as*

$$[\![ x \,|\, \sigma \,|\, \gamma ]\!] = \sigma(x) \qquad [\![ s\,t \,|\, \sigma \,|\, \gamma ]\!] = [\![ s \,|\, \sigma \,|\, \gamma ]\!]\, [\![ t \,|\, \sigma \,|\, \gamma ]\!]$$

$$[\![ c\langle\, c \,\rangle.t \,|\, \sigma \,|\, \gamma ]\!] = \lambda c. [\![ t \,|\, \sigma[c \mapsto c] \,|\, \gamma ]\!]$$

$$[\![ c\langle\, x_1, \dots, x_n \,\rangle.t \,|\, \sigma \,|\, \gamma ]\!] = \lambda c. [\![ t \,|\, \sigma[x_i \mapsto \sigma(x_i)\{c/\gamma(c)\}]_{i \in [n]} \,|\, \gamma ]\!]$$

$$[\![ u[x_1, \dots, x_n \leftarrow t] \,|\, \sigma \,|\, \gamma ]\!] = [\![ u \,|\, \sigma[x_i \mapsto [\![ t \,|\, \sigma \,|\, \gamma ]\!]]_{i \in [n]} \,|\, \gamma ]\!]$$

$$[\![ u[e_1\langle\, \vec{w_1} \,\rangle, \dots, e_n\langle\, \vec{w_n} \,\rangle \,|\, c\langle\, c \,\rangle\, \overline{[\Gamma]}] \,|\, \sigma \,|\, \gamma ]\!] = [\![ u\overline{[\Gamma]} \,|\, \sigma \,|\, \gamma[e_i \mapsto c]_{i \in [n]} ]\!]$$

$$[\![ u[e_1\langle\, \vec{w_1} \,\rangle, \dots, e_n\langle\, \vec{w_n} \,\rangle \,|\, c\langle\, x_1, \dots, x_m \,\rangle\, \overline{[\Gamma]}] \,|\, \sigma \,|\, \gamma ]\!] = [\![ u\overline{[\Gamma]} \,|\, \sigma' \,|\, \gamma[e_i \mapsto c]_{i \in [n]} ]\!]$$

*where* $\sigma' = \sigma[x_i \mapsto \sigma(x_i)\{c/\gamma(c)\}]_{i \in [n]}$

The following Proposition justifies working modulo permutation equivalence.

**Proposition 1.** *For $s, t \in \Lambda_a^S$, if $s \sim t$ then $[\![ s ]\!] = [\![ t ]\!]$.*

### 3.2 Rewrite Rules.

Both the spinal atomic $\lambda$-calculus and the atomic $\lambda$-calculus of [16] follow atomic reduction steps, i.e. they apply on individual constructors. The biggest difference is that our calculus is capable of duplicating not only the skeleton but also the spine. The rewrite rules in our calculus make use of 3 operations, *substitution*, *book-keeping*, and *exorcism*. The operation **substitution** $t\{s/x\}$ propagates through the term $t$, and replaces the free occurences of the variable $x$ with the term $s$. Moreover, if $x$ occurs in the cover of a phantom-variable $e\langle\, \vec{y} \cdot x \,\rangle$, then substitution replaces the $x$ in the cover with $(s)_{fv}$, resulting in $e\langle\, \vec{y} \cdot (s)_{fv} \,\rangle$. Although substitution performs some book-keeping on phantom-abstractions, we define an explicit notion of **book-keeping** $\{\vec{y}/e\}_b$ that updates the variables stored in a free cover i.e. for a term $t$, $e\langle\, \vec{x} \,\rangle \in (t)_{fc}$ then $e\langle\, \vec{y} \,\rangle \in (t\{\vec{y}/e\}_b)_{fc}$. The last operation we introduce is called **exorcism** $\{c\langle\, \vec{x} \,\rangle\}_e$. We perform exorcisms on phantom-abstractions to convert them to abstractions. Intuitively, this will be performed on phantom-abstractions with phantom-variables bound to a distributor when said distributor is eliminated. It converts phantom-abstractions to abstractions by introducing a sharing of the phantom-variable that captures the variables in the cover, i.e. $(c\langle\, \vec{x} \,\rangle.t)\{c\langle\, \vec{x} \,\rangle\}_e = c\langle\, c \,\rangle.t[\vec{x} \leftarrow c]$.

**Proposition 2.** *The translation* $[\![\, u \,|\, \sigma \,|\, \gamma \,]\!]$ *commutes with substitutions, bookkeepings*[1]*, and exorcisms*[2] *in the following way*

$$[\![\, u\{t/x\} \,|\, \sigma \,|\, \gamma \,]\!] = [\![\, u \,|\, \sigma[x \mapsto [\![\, t \,|\, \sigma \,|\, \gamma \,]\!]] \,|\, \gamma \,]\!]$$

$$[\![\, u\{\vec{x}/c\}_b \,|\, \sigma \,|\, \gamma \,]\!] = [\![\, u \,|\, \sigma \,|\, \gamma \,]\!]$$

$$[\![\, u\{c\langle\, x_1, \ldots, x_n \,\rangle\}_e \,|\, \sigma \,|\, \gamma \,]\!] = [\![\, u \,|\, \sigma[x_i \mapsto c]_{i \in [n]} \,|\, \gamma \,]\!]$$

*(1) Given* $c\langle\, \vec{y} \,\rangle \in (u)_{fc}$ *where* $\vec{x} \subseteq \vec{y}$ *and for* $z \in \vec{y}/\vec{x}$, $\gamma(c) \notin (\sigma(z))_{fv}$
*(2) Given* $c\langle\, \vec{x} \,\rangle \in (u)_{fc}$ *or* $\{\vec{x}\} \cap (u)_{fv} = \{\}$

*Proof. See [25], proof of Proposition 18, 19, 20, 21.*

Using these operations, we define the rewrite rules that allow for spinal duplication. Firstly we have beta reduction ($\rightsquigarrow_\beta$), which strictly requires an abstraction (not a phantom).

$(x\langle\, x \,\rangle.t)\, s \rightsquigarrow_\beta t\{s/x\}$

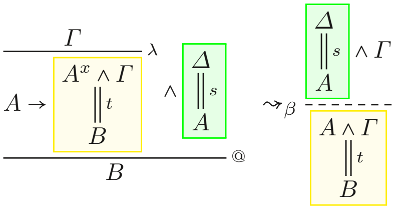

$(\beta)$

Here $\beta$-reduction is a linear operation, since the bound variable $x$ occurs exactly once in the body $t$. Any duplication of the term $t$ in the atomic $\lambda$-calculus proceeds via the sharing reductions.

The first set of sharing reduction rules move closures towards the outside of a term. Most of these rewrite rules only change the typing derivations in the way that subderivations are composed, with the exception of moving a closure out of scope of a distributor.

$$s[\Gamma]\, t \rightsquigarrow_L (s\, t)[\Gamma] \qquad (l_1)$$

$$s\, t[\Gamma] \rightsquigarrow_L (s\, t)[\Gamma] \qquad (l_2)$$

$$d\langle\, \vec{x} \,\rangle.t[\Gamma] \rightsquigarrow_L (d\langle\, \vec{x} \,\rangle.t)[\Gamma] \text{ if } \{\vec{x}\} \cap (t)_{fv} = \{\vec{x}\} \qquad (l_3)$$

$$u[\vec{x} \leftarrow t[\Gamma]] \rightsquigarrow_L u[\vec{x} \leftarrow t][\Gamma] \qquad (l_4)$$

For the case of lifting a closure outside a distributor, we use a notation $\|\, [\Gamma] \,\|$ to identify the variables captured by a closure, i.e. $\|\, [\vec{x} \leftarrow t] \,\| = \{\vec{x}\}$ and $\|\, [e_1\langle\, \vec{x_1} \,\rangle, \ldots, e_n\langle\, \vec{x_x} \,\rangle \,|\, c\langle\, c \,\rangle\, \overline{[\Gamma]}] \,\| = \{\vec{x_1}, \ldots, \vec{x_n}\}$. Then let $\{\vec{z}\} = \|\, [\Gamma] \,\|$ in the following rewrite rule, where we remove $\vec{z}$ from the covers, that can only occur if $\{\vec{x}\} \cap ([\Gamma])_{fv} = \{\}$.

$$\begin{aligned} &u[e_1\langle\, \vec{w_1} \,\rangle \ldots e_n\langle\, \vec{w_n} \,\rangle \,|\, c\langle\, \vec{x} \,\rangle\, \overline{[\Gamma]}][\Gamma]] \\ &\quad \rightsquigarrow_L u\{(\vec{w_i} \setminus \vec{z})/e_i\}_{b_{i \in [n]}}[e_1\langle\, \vec{w_1} \setminus \vec{z} \,\rangle \ldots e_n\langle\, \vec{w_n} \setminus \vec{z} \,\rangle \,|\, c\langle\, \vec{x} \,\rangle\, \overline{[\Gamma]}][\Gamma] \end{aligned} \qquad (l_5)$$

The graphical version of this rule is shown in Figure 4c, where we remove the edge only if there is no edge between $t$ and the unsharing node. The proof rewrite rule corresponding with the rewrite rule $l_5$ can be broken down into two parts. The first part is readjusting how the derivations compose as shown below.

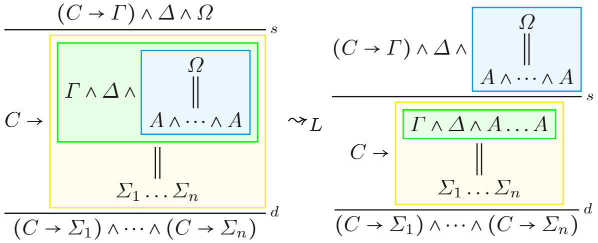

The second part of the rewrite rule justifies the need for the book-keeping operation. In the rewrite below, let $A$ be the type of a variable $z$ where $z \in \vec{z}$. After lifting, we want to remove the variable from the cover as to ensure correctness since the variables in the cover denote the variables captured by the environment. Book-keeping allows us to remove these variables simultaneously.

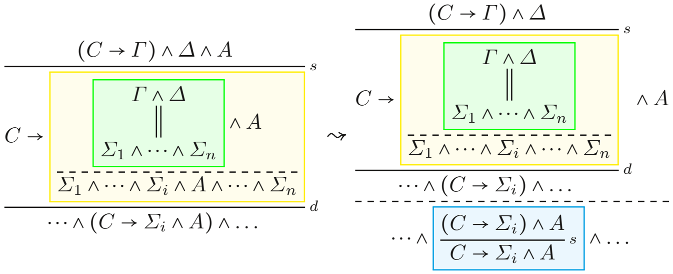

The lifting rules $(l_i)$ are justified by the need to lift closures out of the distributor, as opposed to duplicating them. The second set of rewrite rules, consecutive sharings are compounded and unary sharings are applied as substitutions. For simplicity, in the equivalent proof rewrite step we only show the binary case.

$$u[\vec{w} \leftarrow y][y \cdot \vec{y} \leftarrow t] \rightsquigarrow_C u[\vec{w} \cdot \vec{y} \leftarrow t] \qquad (c_1)$$

$$u[x \leftarrow t] \rightsquigarrow_C u\{t/x\} \qquad (c_2)$$

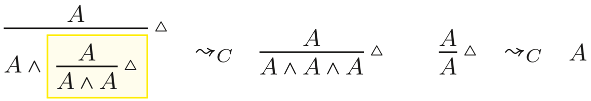

The atomic steps for duplicating are given in the third and final set of rewrite rules. The first being the atomic duplication step of an application, which is the same rule used in [16]. The binary case proof rewrite steps for each rule are also provided. There are also shown graphically in (respectively) Figure 4b (where we maintain links between sharings and unsharings), Figure 4a, and Figure 4d (where the unsharing node is linked to exactly one connecting sharing node).

$$u[x_1 \dots x_n \leftarrow s\,t] \rightsquigarrow_D u\{z_1\,y_1/x_1\}\dots\{z_n\,y_n/x_n\}[z_1 \dots z_n \leftarrow s][y_1 \dots y_n \leftarrow t] \qquad (d_1)$$

$$\dfrac{\dfrac{(A\to B)\wedge A}{B}\,@}{B\wedge B}\,\triangle \quad \rightsquigarrow_D \quad \dfrac{\dfrac{(A\to B)}{(A\to B)\wedge(A\to B)}\,\triangle \wedge \dfrac{B}{B\wedge B}\,\triangle}{\dfrac{(A\to B)\wedge A}{B}\,@ \wedge \dfrac{(A\to B)\wedge A}{B}\,@}$$

$$u[x_1,\ldots,x_n \leftarrow c\langle\,\vec{y}\,\rangle.t] \rightsquigarrow_D$$
$$u\{e_i\langle\,w_i\,\rangle.w_i/x_i\}_{i\in[n]}[e_1\langle\,w_1\,\rangle\ldots e_n\langle\,w_n\,\rangle\,|\,c\langle\,\vec{y}\,\rangle\,[w_1,\ldots,w_n\leftarrow t]] \qquad (d_2)$$

$$\dfrac{\dfrac{(A\to B)\wedge\Gamma}{A\to\boxed{\begin{array}{c}B\wedge\Gamma\\ \Vert\\ C\end{array}}}\,s}{(A\to C)\wedge(A\to C)}\,\triangle \quad \rightsquigarrow_D \quad \dfrac{\dfrac{(A\to B)\wedge\Gamma}{A\to\boxed{\begin{array}{c}B\wedge\Gamma\\ \Vert\\ \dfrac{C}{C\wedge C}\,\triangle\end{array}}}\,s}{(A\to C)\wedge(A\to C)}\,d$$

$$u[e_1\langle\,\vec{w_1}\,\rangle\ldots e_n\langle\,\vec{w_n}\,\rangle\,|\,c\langle\,c\,\rangle\,[\vec{w_1},\ldots,\vec{w_n}\leftarrow c]] \rightsquigarrow_D u\{e_1\langle\,\vec{w_1}\,\rangle\}_e\ldots\{e_n\langle\,\vec{w_n}\,\rangle\}_e \qquad (d_3)$$

$$\dfrac{\dfrac{}{A\to\dfrac{A}{A\wedge A}\,\triangle}\,a}{(A\to A)\wedge(A\to A)}\,d \quad \rightsquigarrow_D \quad \dfrac{}{A\to A}\,a \wedge \dfrac{}{A\to A}\,a$$

*Example 2.* The following example, illustrated in Figure 4e, is a reduction in the term calculus where we duplicate the spine of the term $[a_1, a_2 \leftarrow \lambda x.\lambda y.((\lambda z.z)y)x]$.

$$\rightsquigarrow_D \{x_1\langle b_1\rangle.b_1/a_1\}\{x_2\langle b_2\rangle.b_2/a_2\}[x_1\langle b_1\rangle, x_2\langle b_2\rangle \mid x\langle x\rangle[b_1, b_2\leftarrow\lambda y.((\lambda z.z)y)x]]$$
$$\rightsquigarrow_D \{x_1\langle c_1\rangle.y_1\langle c_1\rangle c_1/a_1\}\{x_2\langle c_2\rangle.y_2\langle c_2\rangle.c_2/a_2\}$$
$$[x_1\langle c_1\rangle, x_2\langle c_2\rangle \mid x\langle x\rangle[y_1\langle c_1\rangle, y_2\langle c_2\rangle \mid y\langle y\rangle[c_1, c_2\leftarrow((\lambda z.z)y)x]]]$$
$$\rightsquigarrow_D \{x_1\langle d_1,e_1\rangle.y_1\langle d_1,e_1\rangle d_1e_1/a_1\}\{x_2\langle d_2,e_2\rangle.y_2\langle d_2,e_2\rangle.d_2e_2/a_2\}$$
$$[x_1\langle d_1,e_1\rangle, x_2\langle d_2,e_2\rangle \mid x\langle x\rangle[y_1\langle d_1,e_1\rangle, y_2\langle d_2,e_2\rangle \mid y\langle y\rangle[d_1,d_2\leftarrow(\lambda z.z)y][e_1,e_2\leftarrow x]]]$$
$$\rightsquigarrow_L \{x_1\langle d_1,e_1\rangle.y_1\langle d_1\rangle d_1e_1/a_1\}\{x_2\langle d_2,e_2\rangle.y_2\langle d_2\rangle.d_2e_2/a_2\}$$
$$[x_1\langle d_1,e_1\rangle, x_2\langle d_2,e_2\rangle \mid x\langle x\rangle[y_1\langle d_1\rangle, y_2\langle d_1\rangle \mid y\langle y\rangle[d_1,d_2\leftarrow(\lambda z.z)y]]\,[e_1,e_2\leftarrow x]]$$
$$\rightsquigarrow_L \{x_1\langle e_1\rangle.y_1\langle d_1\rangle d_1e_1/a_1\}\{x_2\langle e_2\rangle.y_2\langle d_2\rangle.d_2e_2/a_2\}$$
$$[x_1\langle e_1\rangle, x_2\langle e_2\rangle \mid x\langle x\rangle[e_1,e_2\leftarrow x]]\;[y_1\langle d_1\rangle, y_2\langle d_2\rangle \mid y\langle y\rangle[d_1,d_2(\lambda z.z)y]]$$
$$\rightsquigarrow_D \{\lambda x_1.y_1\langle d_1\rangle d_1x_1/a_1\}\{\lambda x_2.y_2\langle d_2\rangle.d_2x_2/a_2\}\;[y_1\langle d_1\rangle, y_2\langle d_2\rangle \mid y\langle y\rangle[d_1,d_2\leftarrow(\lambda z.z)y]]$$

Reduction ($\rightsquigarrow_{(L,C,D,\beta)}$) preserves the conclusion of the derivation, and thus the following proposition is easy to observe.

**Proposition 3.** *If $s \rightsquigarrow_{(L,C,D,\beta)} t$ and $s : A$, then $t : A$.*

**Definition 10.** *For a term $t \in \Lambda_a^S$, if there does not exists a term $s \in \Lambda_a^S$ such that $t \rightsquigarrow_{(L,C,D)} s$ then it is said that $t$ is in **sharing normal form**.*

The following Lemma not only proves we have good translations in Section 3.1, and shows duplication preserves denotation.

**Lemma 1.** *For a* $t \in \Lambda_a^S$ *in sharing normal form and a* $N \in \Lambda$.

$$[\![\, (\!|\, N \,|\!) \,]\!] = N \qquad (\!|\, [\![\, t \,]\!] \,|\!) = t \qquad \exists_{M \in \Lambda}. t = (\!|\, M \,|\!)$$

*Otherwise if* $s \rightsquigarrow_{(L,D,C)} t$ *then* $[\![\, s \,|\, \sigma \,|\, \gamma \,]\!] = [\![\, t \,|\, \sigma \,|\, \gamma \,]\!]$.

*Proof.* See [25, Lemma 24, Lemma 25].

**Lemma 2.** *Given a term* $t \in \Lambda_a^S$, *then* $(\!|\, [\![\, t \,]\!] \,|\!)$ *is* $t$ *in sharing normal form.*

*Proof.* We can prove this by induction on the longest sharing reduction path from $t$. Our base case is already covered by Lemma 1. We are then interested in the inductive case, where $t$ is not in sharing normal form. By Lemma 1, $[\![\, t \,]\!] = [\![\, t' \,]\!]$ where $t \rightsquigarrow_{(D,L,C)} t'$. By induction hypothesis, $(\!|\, [\![\, t' \,]\!] \,|\!)$ is in sharing normal form. Hence $(\!|\, [\![\, t \,]\!] \,|\!)$ is in sharing normal form. □

## 4 Strong Normalisation of Sharing Reductions

In order to show our calculus is strongly normalising, we first show that the sharing reduction rules are strongly normalising. We indite a measure on terms and show that this measure strictly decreases as sharing reduction progresses. Similar ideas and results can be found elsewhere: with *memory* in [20], the *λ-I calculus* in [6], the *λ-void calculus* [2], and the weakening $\lambda\mu$-calculus [17]. Our measure will consist of three components. First, the **height** of a term is a multiset of integers, that measures the number of constructors from each sharing node to the root of the term in its graphical notation. The height is defined on terms as $\mathcal{H}^i(-)$, where $i$ is an integer. We say $\mathcal{H}(t)$ for $\mathcal{H}^1(t)$. We use $\uplus$ to denote the disjoint union of two multisets. We denote $\mathcal{H}^i([\Gamma_1]) \uplus \cdots \uplus \mathcal{H}^i([\Gamma_n])$ as $\mathcal{H}^i(\overline{[\Gamma]})$ for the environment $\overline{[\Gamma]} = [\Gamma_1], \ldots, [\Gamma_n]$.

**Definition 11 (Sharing Height).** *The sharing height* $\mathcal{H}^i(t)$ *of a term* $t$ *is given below, where* $n$ *is the number of closures in* $\overline{[\Gamma]}$*:*

$$\begin{array}{rr}
\mathcal{H}^i(x) = \{\} & \mathcal{H}^i(s\,t) = \mathcal{H}^{i+1}(s) \uplus \mathcal{H}^{i+1}(t) \\
\mathcal{H}^i(c\langle\, \vec{x} \,\rangle.t) = \mathcal{H}^{i+1}(t) & \mathcal{H}^i(t[\Gamma]) = \mathcal{H}^i(t) \uplus \mathcal{H}^i([\Gamma]) \uplus \{i^1\} \\
\mathcal{H}^i([x_1, \ldots, x_n \leftarrow t]) = \mathcal{H}^{i+1}(t) & \mathcal{H}^i([\vec{w} \,|\, c\langle\, \vec{x} \,\rangle\, \overline{[\Gamma]}]) = \mathcal{H}^{i+1}(\overline{[\Gamma]}) \uplus \{(i+1)^n\}
\end{array}$$

This measure then strictly decreases for the rewrite rules $l_1$, $l_2$, $l_3$, $l_4$ and $l_5$, i.e. if $t \rightsquigarrow_L u$ then $\mathcal{H}^i(t) > \mathcal{H}^i(u)$. The second measure we consider is the **weight** of a term. Intuitively this quantifies the remaining duplications, which are performed with $\rightsquigarrow_D$ reductions. If a term would be deleted, we assign it with a weight '1' to express that it is not duplicated. Calculating the weight requires an auxiliary function that assigns integer weights to the variables of a term. This function is defined on terms $\mathcal{V}^i(-)$, where $i$ is an integer. To measure variables independently of binders is vital. It allows to measure distributors, which duplicate $\lambda$'s but not the bound variable. Also, only bound variables for abstractions are measured since variables bound by sharings are substituted in the interpretation.

**Definition 12 (Variable Weights).** *The function $\mathcal{V}^i(t)$ returns a function that assigns integer weights to the free variables of $t$. It is defined by the below, where $f = \mathcal{V}^i(t)$ and $g = f(x_1) + \cdots + f(x_n)$ for each $x_i \in \vec{x}$.*

$$\mathcal{V}^i(x) = \{x \mapsto i\} \qquad \mathcal{V}^i(s\,t) = \mathcal{V}^i(s) \uplus \mathcal{V}^i(t)$$
$$\mathcal{V}^i(c\langle\, c\,\rangle.t) = \mathcal{V}^i(t)/\{c\} \qquad \mathcal{V}^i(c\langle\, \vec{x}\,\rangle.t) = \mathcal{V}^i(t) \uplus \{c \mapsto i\}$$
$$\mathcal{V}^i(t[\vec{x} \leftarrow s]) = \mathcal{V}^i(t)/\{\vec{x}\} \uplus \mathcal{V}^g(s) \qquad \mathcal{V}^i(t[\leftarrow s]) = \mathcal{V}^i(t) \uplus \mathcal{V}^1(s)$$
$$\mathcal{V}^i(t[e_1\langle\, \vec{w}_1\,\rangle \ldots e_n\langle\, \vec{w}_n\,\rangle \,|\, c\langle\, c\,\rangle\, \overline{[\Gamma]}]) = \mathcal{V}^i(t\overline{[\Gamma]})/\{c, e_1, \ldots, e_n\}$$
$$\mathcal{V}^i(t[e_1\langle\, \vec{w}_1\,\rangle \ldots e_n\langle\, \vec{w}_n\,\rangle \,|\, c\langle\, \vec{x}\,\rangle\, \overline{[\Gamma]}]) = \mathcal{V}^i(t\overline{[\Gamma]})/\{e_1, \ldots, e_n\} \uplus \{c \mapsto i\}$$

The weight of a term can then be defined via the use of this auxiliary function. The auxiliary function is used when calculating the weight of a sharing, where the sharing weight of the variables bound by the sharing play a significant role in calculating the weight of the shared term. In the case of a weakening $[\leftarrow t]$, we assign an initial weight of 1. Again we say $\mathcal{W}(t) = \mathcal{W}^1(t)$.

**Definition 13 (Sharing Weight).** *The sharing weight $\mathcal{W}^i(t)$ of a term $t$ is a multiset of integers computed by the function defined below, where $f = \mathcal{V}^i(t)$ and $g = f(x_1) + \cdots + f(x_n)$ for each $x_i \in \vec{x}$.*

$$\mathcal{W}^i(x) = \{\} \qquad \mathcal{W}^i(s\,t) = \mathcal{W}^i(s) \uplus \mathcal{W}^i(t) \uplus \{i\}$$
$$\mathcal{W}^i(c\langle\, c\,\rangle.t) = \mathcal{W}^i(t) \uplus \{i\} \uplus \{\mathcal{V}^i(t)(c)\} \qquad \mathcal{W}^i(c\langle\, \vec{x}\,\rangle.t) = \mathcal{W}^i(t) \uplus \{i\}$$
$$\mathcal{W}^i(t[\vec{x} \leftarrow s]) = \mathcal{W}^i(t) \uplus \mathcal{W}^g(s) \qquad \mathcal{W}^i(t[\leftarrow s]) = \mathcal{W}^i(t) \uplus \mathcal{W}^1(s)$$
$$\mathcal{W}^i(t[e_1\langle\, \vec{w}_1\,\rangle \ldots e_n\langle\, \vec{w}_n\,\rangle \,|\, c\langle\, c\,\rangle\, \overline{[\Gamma]}]) = \mathcal{W}^i(t\overline{[\Gamma]}) \uplus \{\mathcal{V}^i(t\overline{[\Gamma]})(c)\}$$
$$\mathcal{W}^i(t[e_1\langle\, \vec{w}_1\,\rangle \ldots e_n\langle\, \vec{w}_n\,\rangle \,|\, c\langle\, \vec{x}\,\rangle\, \overline{[\Gamma]}]) = \mathcal{W}^i(t\overline{[\Gamma]})$$

This measure then strictly decreases on the rewrite rules $d_1$, $d_2$, $d_3$ and is unaffected by all the other sharing reduction rules, i.e. if $t \rightsquigarrow_D u$ then $\mathcal{W}^i(t) > \mathcal{W}^i(u)$. If $t \rightsquigarrow_{(L,C)} u$ then $\mathcal{W}^i(t) = \mathcal{W}^i(u)$. The third and last measure we consider is the **number of closures** in the term, where it can be easily observed that the rewrite rules $c_1$ and $c_2$ strictly decrease this measure, and that the $\rightsquigarrow_L$ rules do not alter the number of closures. We then use this along with height and weight to define a *sharing measure* on terms.

**Definition 14.** *The **sharing measure** of a $\Lambda^S_a$-term $t$ is a triple $(\mathcal{W}(t), \mathcal{C}, \mathcal{H}(t))$, where $\mathcal{C}$ is the number of closures in the term $t$. We compare sharing measures by using the lexicographical preferences according to $\mathcal{W} > \mathcal{C} > \mathcal{H}$.*

**Theorem 1.** *Sharing reduction $\rightsquigarrow_{(D,L,C)}$ is strongly normalising.*

Now that we have proven the sharing reductions are strongly normalising, we can prove that they are confluent for closed terms.

**Theorem 2.** *The sharing reduction relation $\rightsquigarrow_{(D,L,C)}$ is confluent.*

*Proof.* Lemma 1 tells us that the preservation is preserved under reduction i.e. for $s \rightsquigarrow_{(D,L,C)} t$, $[\![ s ]\!] = [\![ t ]\!]$. Therefore given $t \rightsquigarrow^*_{(D,L,C)} s_1$ and $t \rightsquigarrow^*_{(D,L,C)} s_2$, $[\![ t ]\!] = [\![ s_1 ]\!] = [\![ s_2 ]\!]$. Since we know that sharing reductions are strongly normalising, we know there exists terms $u_1$ and $u_2$ in sharing normal form such that $s_1 \rightsquigarrow^*_{(D,L,C)} u_1$ and $s_2 \rightsquigarrow^*_{(D,L,C)} u_2$. Lemma 1 tells us that terms in sharing normal form are in correspondence with their denotations i.e. $(\!| [\![ t ]\!] |\!) = t$. Since by Lemma 1 we know $[\![ u_1 ]\!] = [\![ s_1 ]\!] = [\![ s_2 ]\!] = [\![ u_2 ]\!]$, and by Lemma 1 $(\!| [\![ u_1 ]\!] |\!) = u_1$ and $(\!| [\![ u_2 ]\!] |\!) = u_2$, we can conclude $u_1 = u_2$. Hence, we prove confluence. □

## 5 Preservation of Strong Normalisation and Confluence

A $\beta$-step in our calculus may occur within a weakening, and therefore is simulated by zero $\beta$-steps in the $\lambda$-calculus. Therefore if there is an infinite reduction path located inside a weakening in $\Lambda_a^S$, then the reduction path is not preserved in the corresponding $\lambda$-term as there are no weakenings. To deal with this, just as done in [2, 16, 17], we make use of the **weakening calculus**. A $\beta$-step is non-deleting precisely because of the weakening construct. If a $\beta$-step would be deleting, then the weakening calculus would instead keep the deleted term around as 'garbage', which can continue to reduce unless explicitly 'garbage-collected' by extra (non-$\beta$) reduction steps. PSN has already be shown for the weakening calculus through the use of a perpetual strategy in [16]. A part of proving PSN is then using the weakening calculus to prove that if $t \in \Lambda_a^S$ has a infinite reduction path, then its translation into the weakening calculus also has an infinite reduction path.

**Definition 15.** *The $\mathcal{W}$-terms of the weakening calculus ($\Lambda_{\mathcal{W}}$) are*

$$T, U, V \quad ::= \quad x \quad | \quad \lambda x.T^* \quad | \quad U\,V \quad | \quad T[\leftarrow U] \quad | \quad \bullet \text{ (*) where } x \in (T)_{fv}$$

The terms are variable, abstraction, application, weakening, and a bullet. In the weakening $T[\leftarrow U]$, the subterm $U$ is *weakened*. The interpretation of atomic terms to weakening terms $[\![ - | - | - ]\!]_{\mathcal{W}}$ can be seen as an extension of the translation into the $\lambda$-calculus (Definition 9).

**Definition 16.** *The interpretation $[\![ - | - | - ]\!]_{\mathcal{W}} : \Lambda_a^S \times (V \to \Lambda_{\mathcal{W}}) \times (V \to V) \to \Lambda_{\mathcal{W}}$ with maps $\sigma : V \to \Lambda_{\mathcal{W}}$ and $\gamma : V \to V$ is defined as an extension of the translation in (Definition 9) with the following additional special cases.*

$$\begin{aligned} [\![ u[\leftarrow t] \,|\, \sigma \,|\, \gamma ]\!]_{\mathcal{W}} &= [\![ u \,|\, \sigma \,|\, \gamma ]\!]_{\mathcal{W}}[\leftarrow [\![ t \,|\, \sigma \,|\, \gamma ]\!]_{\mathcal{W}}] \\ [\![ u[\,|\, c\langle\, c \,\rangle\, \overline{[\Gamma]}] \,|\, \sigma \,|\, \gamma ]\!]_{\mathcal{W}} &= [\![ u\overline{[\Gamma]} \,|\, \sigma[c \mapsto \bullet] \,|\, \gamma ]\!]_{\mathcal{W}} \\ [\![ u[\,|\, c\langle\, x_1, \ldots, x_n \,\rangle\, \overline{[\Gamma]}] \,|\, \sigma \,|\, \gamma ]\!]_{\mathcal{W}} &= [\![ u\overline{[\Gamma]} \,|\, \sigma' \,|\, \gamma ]\!]_{\mathcal{W}} \end{aligned}$$

*where* $\sigma'(z) := \; \textit{if } z \in \{x_1, \ldots, x_n\} \textit{ then } \sigma(z)\{\bullet/\gamma(c)\} \textit{ else } \sigma(z)$

We say $[\![ t ]\!]^{\mathcal{W}} = [\![ t \,|\, I \,|\, I ]\!]_{\mathcal{W}}$ where $I$ is the identity function. We also have translations of the weakening calculus to and from the $\lambda$-calculus. Both of these translations were provided in [16]. The interpretation $\lfloor - \rfloor$ from weakening terms to $\lambda$-terms discards all weakenings.

**Definition 17.** *The interpretation $M \in \Lambda$, $(\!| - |\!)^{\mathcal{W}} : \Lambda \to \Lambda_{\mathcal{W}}$ is defined below.*

$$(\!| x |\!)^{\mathcal{W}} = x \quad (\!| M\,N |\!)^{\mathcal{W}} = (\!| M |\!)^{\mathcal{W}}\,(\!| N |\!)^{\mathcal{W}} \quad (\!| \lambda x.N |\!)^{\mathcal{W}} = \begin{cases} \lambda x.(\!| N |\!)^{\mathcal{W}} & \text{if } x \in (N)_{fv} \\ \lambda x.(\!| N |\!)^{\mathcal{W}}[\leftarrow x] & \text{otherwise} \end{cases}$$

The following equalities can be observed, where $\sigma^{\Lambda}(z) = \lfloor \sigma^{\mathcal{W}}(z) \rfloor$.

**Proposition 4.** *For $N \in \Lambda$ and $t \in \Lambda_a^S$ the following properties hold*

$$\lfloor [\![ t \,|\, \sigma^{\mathcal{W}} \,|\, \gamma ]\!]_{\mathcal{W}} \rfloor = [\![ t \,|\, \sigma^{\Lambda} \,|\, \gamma ]\!] \qquad [\![ (\!| N |\!) ]\!]^{\mathcal{W}} = (\!| N |\!)^{\mathcal{W}} \qquad \lfloor (\!| N |\!)^{\mathcal{W}} \rfloor = N$$

*where for each $\{x \mapsto M\} \in \sigma^{\mathcal{W}}$, $\{x \mapsto \lfloor M \rfloor\} \in \sigma^{\Lambda}$.*

**Definition 18.** *In the weakening calculus, $\beta$-reduction is defined as follows, where $\overline{[\Gamma]}$ are weakening constructs. $((\lambda x.T)\overline{[\Gamma]})\,U \to_\beta T\{U/x\}\overline{[\Gamma]}$*

**Proposition 5.** *If $N \in \Lambda$ is strongly normalising, then so is $(\!| N |\!)^{\mathcal{W}}$.*

When translating from $\Lambda_a^S$ to $\Lambda_{\mathcal{W}}$, weakenings are maintained whilst sharings are interpreted via substitution. Thus the reduction rules in the weakening calculus cover the spinal reductions for nullary distributors and weakenings.

**Definition 19.** *Weakening reduction ($\to_{\mathcal{W}}$) proceeds as follows.*

$$\begin{array}{rlrl} U[\leftarrow T]\,V & \to_{\mathcal{W}} (U\,V)[\leftarrow T] & U\,V[\leftarrow T] & \to_{\mathcal{W}} (U\,V)[\leftarrow T] \\ T[\leftarrow U[\leftarrow V]] & \to_{\mathcal{W}} T[\leftarrow U][\leftarrow V] & T[\leftarrow \lambda x.U] & \to_{\mathcal{W}} T[\leftarrow U\{\bullet/x\}] \\ T[\leftarrow U\,V] & \to_{\mathcal{W}} T[\leftarrow U][\leftarrow V] & T[\leftarrow \bullet] & \to_{\mathcal{W}} T \\ T[\leftarrow U] & \to_{\mathcal{W}} T^{(1)} & \lambda x.T[\leftarrow U] & \to_{\mathcal{W}} (\lambda x.T)[\leftarrow U]^{(2)} \end{array}$$

*(1) if $U$ is a subterm of $T$ and (2) if $x \notin (U)_{fv}$*

It is easy to see that these rules correspond to special cases of the sharing reduction rules for $\Lambda_a^S$. This resemblance is confirmed by the following Lemma, proven in [25, pp. 82-86]. We use this to show how $\Lambda_a^S$ enjoys PSN.

**Lemma 3.** *If $t \rightsquigarrow_\beta u$ then $[\![ t ]\!]^{\mathcal{W}} \to_\beta^+ [\![ u ]\!]^{\mathcal{W}}$. If $t \rightsquigarrow_{(C,D,L)} u$ and for any $x \in (t)_{bv} \cup (t)_{fp}$ such that for all $z$, $x \notin (\sigma(z))_{fv}$.*

$$[\![ t \,|\, \sigma \,|\, \gamma ]\!]_{\mathcal{W}} \to_{\mathcal{W}}^* [\![ u \,|\, \sigma \,|\, \gamma ]\!]_{\mathcal{W}}$$

**Lemma 4.** *For $t \in \Lambda_a^S$ has an infinite reduction path, then $[\![ t ]\!]^{\mathcal{W}}$ also has an infinite reduction path.*

*Proof.* Due to Theorem 2, we know that the infinite reduction path contains infinite $\beta$-steps. This means in the reduction sequence, between each $\beta$-step, there are finite many $\rightsquigarrow_{(D,L,C)}$ reduction steps. Lemma 3 says each $\rightsquigarrow_{(D,L,C)}$ step in $\Lambda_a^S$ corresponds to zero or more weakening reductions ($\rightsquigarrow_{\mathcal{W}}^*$). Lemma 3 says that each beta step in $\Lambda_a^S$ corresponds to one or more $\beta$-steps in $\Lambda_{\mathcal{W}}$. Therefore, it must be that $[\![ t ]\!]^{\mathcal{W}}$ also has an infinite reduction path. □

**Theorem 3.** *If $N \in \Lambda$ is strongly normalising, then so is $(\!| N |\!)$.*

*Proof.* For a given $N \in \Lambda$ that is strongly normalising, we know by Lemma 5 that $(\!| N |\!)^{\mathcal{W}}$ is strongly normalising. Then $[\![ (\!| N |\!) ]\!]^{\mathcal{W}}$ is strongly normalising, since Proposition 4 states that $(\!| N |\!)^{\mathcal{W}} = [\![ (\!| N |\!) ]\!]^{\mathcal{W}}$. Then by Lemma 4, which states that if $[\![ t ]\!]^{\mathcal{W}}$ is strongly normalising, then $t$ is strongly normalising, proves that $(\!| N |\!)$ is strongly normalising. □

We also prove confluence, which is already known for the $\lambda$-calculus [11]. We first observe that a $\beta$-step in the $\lambda$-calculus is simulated in $\Lambda_a^S$ by one $\beta$-step followed by zero or more sharing reductions.

**Lemma 5.** *Given $N, M \in \Lambda$. If $N \rightsquigarrow_\beta M$, then $(\!| N |\!) \rightsquigarrow_\beta \rightsquigarrow^*_{(D,L,C)} (\!| M |\!)$.*

*Proof.* This is proven by Sherratt in [25, Lemma 67].

**Theorem 4.** *Given $t, s_1, s_2 \in \Lambda_a^S$. If $t \rightsquigarrow^*_{(\beta,D,L,C)} s_1$ and $t \rightsquigarrow^*_{(\beta,D,L,C)} s_2$, there exists a $u \in \Lambda_a^S$ such that $s_1 \rightsquigarrow^*_{(\beta,D,L,C)} u$ and $s_2 \rightsquigarrow^*_{(\beta,D,L,C)} u$.*

*Proof.* Suppose $t \rightsquigarrow^*_{(\beta,D,L,C)} s_1$ and $t \rightsquigarrow^*_{(\beta,D,L,C)} s_2$. Then we have $[\![ t ]\!] \rightsquigarrow^*_\beta [\![ s_1 ]\!]$ and $[\![ t ]\!] \rightsquigarrow^*_\beta [\![ s_2 ]\!]$. By the Church-Rosser theorem, there exists a $M \in \Lambda$ such that $[\![ s_1 ]\!] \rightsquigarrow^*_\beta M$ and $[\![ s_2 ]\!] \rightsquigarrow^*_\beta M$. Due to Lemma 2, $(\!| [\![ s_1 ]\!] |\!) = s_1'$ and $(\!| [\![ s_2 ]\!] |\!) = s_2'$ where $s_1', s_2' \in \Lambda_a^S$ in sharing normal form. Then thanks to Lemma 5 we know $s_1' \rightsquigarrow^*_{(\beta,D,L,C)} (\!| M |\!)$ and $s_2' \rightsquigarrow^*_{(\beta,D,L,C)} (\!| M |\!)$. Combined, we get confluence. □

## 6 Conclusion, related work, and future directions

We have studied the interaction between the switch and the medial rule, the two characteristic inference rules of deep inference. We built a Curry–Howard interpretation based on this interaction, whose resulting calculus not only has the ability to duplicate terms atomically but can also duplicate solely the spine of an abstraction such that beta reduction can proceed on the duplicates. We show that this calculus has natural properties with respect to the $\lambda$-calculus.

This work, which started as an investigation into the Curry-Howard correspondence of the switch rule [25], fits into a broader effort to give a computational interpretation to intuitionistic deep-inference proof theory. Brünnler and McKinley [9] give a natural reduction mechanism without medial (or switch), and observe that preservation of strong normalization fails. Guenot and Straßburger [14] investigate a different switch rule, corresponding to the implication-left rule of sequent calculus. He [17] extends the atomic $\lambda$-calculus to the $\lambda\mu$-calculus.

Our future goal is to develop the intuitionistic open deduction formalism towards optimal reduction [23, 21, 3], via the remaining medial and switch rules [26].

**Acknowledgements** We thank the anonymous reviewers for their comments.
